# Therapeutic Nanoparticles for the Different Phases of Ischemic Stroke

**DOI:** 10.3390/life11060482

**Published:** 2021-05-26

**Authors:** Sara Bernardo-Castro, Inês Albino, Ángela María Barrera-Sandoval, Francesca Tomatis, João André Sousa, Emanuel Martins, Susana Simões, Miguel M. Lino, Lino Ferreira, João Sargento-Freitas

**Affiliations:** 1 Neurology Department, Centro Hospitalar e Universitário de Coimbra, 3004-561 Coimbra, Portugal; 15348@chuc.min-saude.pt (S.B.-C.); 11472@chuc.min-saude.pt (J.A.S.); emanuelmartins@chuc.min-saude.pt (E.M.); 2 Faculty of Medicine, University Coimbra, 3000-548 Coimbra, Portugal; angelamb.sandoval@cnc.uc.pt (A.M.B.-S.); lino@uc-biotech.pt (L.F.); 3 IIIUC-Institute of Interdisciplinary Research, University of Coimbra, Casa Costa Alemão, 3030-789 Coimbra, Portugal; ialbino@cnc.uc.pt (I.A.); ftomatis@cnc.uc.pt (F.T.); susana.simoes@cnc.uc.pt (S.S.); miguel.lino@cnc.uc.pt (M.M.L.); 4 CNC-Center for Neuroscience and Cell Biology, CIBB-Centre for Innovative Biomedicine and Biotechnology, University of Coimbra, UC, Biotech Parque Tecnológico de Cantanhede, 3060-197 Coimbra, Portugal; 5 PhD Programme in Experimental Biology and Biomedicine, Institute for Interdisciplinary Research (IIIUC), University of Coimbra, Casa Costa Alemão, 3030-789 Coimbra, Portugal

**Keywords:** stroke, nanoparticles, stroke phases, ischemia

## Abstract

Stroke represents the second leading cause of mortality and morbidity worldwide. Ischemic strokes are the most prevalent type of stroke, and they are characterized by a series of pathological events prompted by an arterial occlusion that leads to a heterogeneous pathophysiological response through different hemodynamic phases, namely the hyperacute, acute, subacute, and chronic phases. Stroke treatment is highly reliant on recanalization therapies, which are limited to only a subset of patients due to their narrow therapeutic window; hence, there is a huge need for new stroke treatments. Nonetheless, the vast majority of promising treatments are not effective in the clinical setting due to their inability to cross the blood-brain barrier and reach the brain. In this context, nanotechnology-based approaches such as nanoparticle drug delivery emerge as the most promising option. In this review, we will discuss the current status of nanotechnology in the setting of stroke, focusing on the diverse available nanoparticle approaches targeted to the different pathological and physiological repair mechanisms involved in each of the stroke phases.

## 1. Introduction

Stroke affects one in four people over their lifetime and is a leading cause of death and disability in adults worldwide [1]. Stroke is defined as a neurological deficit due to an acute focal injury of the central nervous system by a vascular cause [2]. Current acute treatments for ischemic stroke include intravenous administration of human tissue plasminogen activator (intravenous thrombolysis) and endovascular thrombectomy. Intravenous thrombolysis (IVT) aims to cleave the thrombus causing the stroke by enzymatic means. This treatment is applied during the first 4.5 h after stroke onset [2] while the endovascular thrombectomy (EVT) has high success rates in patients with stroke due to large vessel occlusions and can be used in selected patients up to 24 h after onset, albeit its efficacy is also very time-dependent [3]. This treatment is performed in an angiography suite, where a catheter is inserted in an artery by a specialized physician, monitored by X-ray imaging until reaching the blood clot and removing it. Unfortunately, in many cases, patients who survive a stroke event have limited functional recovery due to an incomplete remodeling and restorative process in the lesion area. Neuroprotective strategies targeting the cascade of cellular and molecular events that lead to ischemic damage, and strategies to promote post-ischemic regeneration, have been pursued in the last years, although clinical translation has not been fulfilled yet.

In recent years, several advanced diagnostic and therapeutic applications have been proposed based on new pharmaceutical entities, cell-based therapies, and biomaterials [4,5]. One of the strategies that has attracted much attention is based on the use of nanoparticles (NPs) for diagnostic and therapeutic applications [6]. The motivation here is to increase the lifespan of therapeutics in the bloodstream and to enhance their permeation through the blood-brain barrier (BBB) to reach the ischemic site. Most of these strategies have been used only in pre-clinical animal models and thus have not reached clinical use. Although the use of NPs in the context of stroke has been the focus of recent reviews [7,8,9], the use of NPs to target specific requirements of the different phases of ischemic stroke has not been covered.

**Figure 1:**
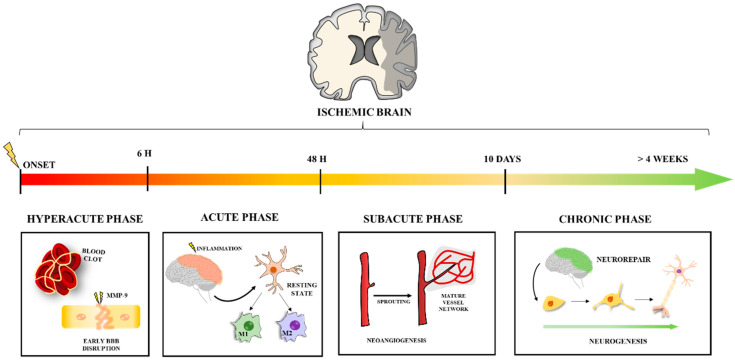
Main processes to be targeted in each phase of ischemic stroke. Stroke follows a time-course progression throughout different phases with distinct underlying mechanisms that can be targeted to improve recovery. Blood clot formation and early BBB disruption are the key points to be targeted in the hyperacute phase. The acute phase occurs thereafter, with neuroinflammation as the main factor in injury development. Microglia activation to M1 and/or M2 phenotypes is one of the main processes for NP targeting. The subacute and chronic phases characterize repair processes, mainly neoangiogenesis and neurogenesis, respectively. Promoting this neurorepair mechanism is the main focus for NP targeting in these final phases.

In the present review, we describe the use of NPs in stroke management according to the different stroke phases (Figure 1). We provide a deep understanding of the molecular mechanisms that can be tackled with different types of NPs. This work aims to provide an overview of different nanotechnology-based approaches to treat the complex cellular and molecular mechanisms that lead to the pathophysiological response in ischemic stroke. This knowledge will hopefully offer guidance on better target selection for stroke treatment and on what is currently lacking for an effective translation into the clinics.

## 2. Stroke: Physiopathology and Treatment Limitations

Stroke can be divided into two different types: ischemic and hemorrhagic. Ischemic stroke is caused by interruption of blood supply to a part of the brain resulting in a sudden loss of function usually caused by an occluded artery, while hemorrhagic stroke is attributed to rupture of a blood vessel or an abnormal vascular structure [10]. Generally, acute ischemic strokes (AIS) account for about 80% of all stroke cases and will be the main focus of this review, particularly considering the distinct mechanisms involved in these two cerebrovascular diseases [10,11]. Due to its prevalence, mainly in older populations, stroke prevention and treatment has arisen as a major issue in the health planning agenda, with one of the biggest advances being the implementation of regional and national acute-phase stroke networks, that allows for fast intervention when, during a stroke, time is critical.

BBB disruption is one of the main pathophysiological features of stroke [12], and hence, understanding the dynamics of BBB disruption and repair is of particular importance for the prevention of undesirable outcomes such as hemorrhagic transformation (HT) on AIS. The BBB is a dynamic physiological structure that constitutes an interface between the vasculature system and the neural tissues maintaining its homeostasis while preventing unwanted compounds from entering the brain [13]. After an AIS, the BBB undergoes different hemodynamic phases where its permeability (BBBP) increase can favor undesirable outcomes such as HT on the one hand or enhance neoangiogenesis allowing the delivery of potentially therapeutic agents on the other hand. Each of the hemodynamic phases, namely hyperacute (<6 h), acute (6–72 h), subacute (>72 h), and chronic phase (>4 weeks), have their own processes, with different pathological responses and often opposing clinical consequences that need to be addressed [4]. In short, the hyperacute phase is characterized by a first BBB disruption and cell death due to the sudden hypoxia [14]. During the next 72/96 h, in the acute phase, the neuroinflammation processes motivated by the first cytotoxic events will further rupture the BBB, leading to immune cells infiltration into the brain [15]. Around one week after stroke onset, the subacute phase takes place. This phase is marked by the start of recovering processes such as angiogenesis [16]. While BBB has been proven to still be permeable at this point, this permeability is believed to be beneficial for clinical outcomes [17]. In the late phase of cerebral ischemic injury, the chronic phase, neurogenesis, and other neuroregenerative processes such as vasculogenesis usually occur along with restoration, at least partially, of the BBB integrity [12,16]. Stroke treatment from the hyperacute phase until rehabilitation is paramount. The mainstay of hyperacute and acute-phase treatment of ischemic stroke is still recanalization with intravenous or intra-arterial therapies. Intravenous thrombolysis (IVT) through tissue plasminogen activator (tPA), a clot-dissolving drug, is widely used due to its accessibility and demonstrated ability to reduce stroke disability. However, eligibility for IVT, due to its time-limited benefit, restricts its effective administration for the majority of stroke patients. In recent years due to the development of endovascular mechanical thrombectomy (EVT), a catheter-based image-guided intervention for the mechanical removal of blood clots in large brain arteries, prognosis has changed dramatically [18], and eligibility for acute-phase treatment has widened due to fewer time constraints and fewer contraindications [18]. Unfortunately, several factors still limit the use of recanalization therapies to a minority of AIS patients [19,20] and over half of stroke survivors undergoing these therapies still have poor functional outcomes [21]. Treatments for stroke care in the subacute stage are limited to a strategy of neuroprotection exclusively through the blood pressure, fluid volume, glycemia and oxygen control in order to avoid secondary damage. Lastly, in the chronic stage, pharmacologically, we can only target secondary prevention with antiplatelets or oral anticoagulants, depending on the stroke etiology, which remains unclear in one-fourth of patients [22]. Rehabilitation is achieved with more or less success through activity-based therapies (physiotherapy), but most patients live with enduring disabilities [23]. Therefore, while AIS care can be effective in reducing infarct size and reversing neurologic deficits through reperfusion and recanalization, we are still devoid of therapeutic drugs or directed strategies to make brain cells more tolerable to ischemia or to dampen the pathological processes that persist after the acute ischemic insult, including inflammation, excitotoxicity, oxidative stress, apoptosis, and edema resulting from BBB disruption [4,24]. Moreover, subacute promotion of brain plasticity and neurorepair aiming to restore the anoxic lost core of brain tissue after stroke has also not yet reached bedside medicine. In fact, there are currently no approved pharmacological treatments with neuroprotective or neuroregenerative actions [25]. In this context, NPs have the potential to overcome many of the limitations stroke medicine currently faces in clinical practice. These include, in the hyperacute and acute phases, the risk of hemorrhagic transformation associated with recanalization therapies that may range from 5% to 30% [21,26,27,28] and the already mentioned inexistence of therapies that enhance either neoangiogenesis that peaks in the subacute phase and is associated with better clinical outcomes [17], or neurorepair/neuroregeneration that persists throughout the chronic phase of an ischemic stroke.

## 3. NPs: Composition and Properties

NPs can be described as material in which at least 50% of their particles have at minimum one dimension in the size range of 1–100 nm [29]. Broadly, NPs can be classified into two major groups: synthetic and biological. Among the biological NPs, we can find extracellular vesicles (EVs). EVs are biological NPs secreted by cells that contain biomolecules (miRNAs, proteins, lipids) able to modulate cell activity at a distance [30]. Several studies have tested EVs in the last 5 years in the context of stroke [31]. On the other side, synthetic NPs comprise a large set of nanomaterials, including nanocapsules, nanogels, liposomes, and micelles, among others. These NPs share novel and unique physicochemical properties that differ from those of bulk materials, representing a whole new set of opportunities in drug development [32]. Their unique small size and large surface area to volume ratio make the carried therapeutic compounds to be closer to the surface of the NPs, leading to a faster rate of drug release and higher bioavailability [32]. Moreover, targeted and controlled delivery through NPs protects the therapeutic compounds from deactivation and clearance and improves their pharmacodynamics and safety while preventing off-site interactions [33].

**Figure 2:**
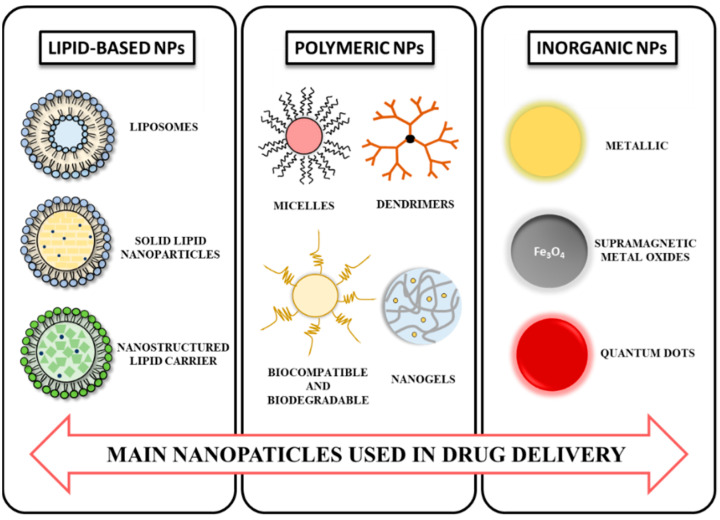
Main therapeutic synthetic NPs used in drug delivery. Synthetic NPs can be broadly divided into lipid-based, polymeric, and inorganic NPs. The figure represents the main NPs studied in stroke care in each of the categories.

A vast variety of synthetic NPs are currently available, and their classification can be made according to different criteria such as dimensionality, morphology, state, or chemical composition [34]. Mainly, therapeutic synthetic NPs can be divided into three categories [35]: (1) lipid-based NPs, (2) polymeric NPs [36], and (3) inorganic and metallic NPs (Figure 2). Several types of lipid-based NPs have been approved by regulatory agencies for clinical use. These NPs are able to transport both hydrophobic and hydrophilic molecules protected by lipids, display low toxicity and significantly increase drug bioavailability [37]. Lipid-based NPs include liposomes, spherical vesicles composed of phospholipids and steroids, bilayers, or other surfactants [38]; solid lipid nanoparticles (SLN), made of solid lipids stabilized by various surfactants with good physical stability and tolerability [39]; non-structured lipid carriers (NLC) composed by a mixture of solid and liquid lipids, which leads to a special nanostructure with increased payload [38] and lipid drug conjugates (LDC). On its side, polymeric NPs can be obtained from either synthetic or natural polymers [37] and are assembled in nanoformulations with different sizes and shapes. Polymeric NPs include micelles, [40] dendrimers, [41] and polymeric nanogels [42], among others [43,44]. Finally, inorganic NPs include metal NPs [45]; metal oxides, which possess superparamagnetic properties and are useful as contrast agents; quantum dots, typically made of semiconducting materials [35] and ceramic NPs.

The surface of any NP is a key component for its properties. In fact, despite their intrinsic characteristics, NPs normally lack selective distribution across the body, and as soon as they enter the bloodstream, they are prone to aggregation and protein opsonization and therefore can be rapidly cleared from the body, resulting in decreased retention time and thus limited bioavailability [35]. To overcome this, their surface is usually coated with molecules such as polymers, small molecules, peptides, or proteins that prevent the adsorption of proteins to their surface and simultaneously enhancing their capacity to interact with a specific cell/tissue target [46]. For example, NPs coated with polysorbate 80 [47] or polyethylene glycol (PEG) [48] showed prolonged circulation in the bloodstream and different body distribution as non-coated ones. In addition, functionalization by antibodies [49] or proteins such as lactoferrin [50] allow specific interactions between the NP and the target.

## 4. NPs in Stroke Diagnosis

NPs are useful in molecular brain imaging to reveal biological processes that constitute potential diagnostic or therapeutic targets in stroke [9]. High-resolution imaging of the cerebral vasculature is a major goal of stroke research, and as such, nanotechnology-based brain imaging has the potential to allow a much more detailed picture of the extent of the ischemic injury [51].

The main imaging tools in stroke diagnosis are magnetic resonance imaging (MRI) and computed tomography (CT) [7,52]. These imaging tools are relevant to detect the early phase of stroke, to evaluate the most relevant pathological characteristics of stroke such as BBB disruption, and to stratify the patients that can benefit from tPA recanalization therapy, among others. For example, iron oxide NPs have been used as a contrast agent for early detection of neuroinflammation in ischemic stroke by MRI [53], while α_v_β_3_integrin-targeted NPs have been used successfully to monitor pro-angiogenic response in ischemic stroke, particularly in diabetic animal models [54]. Moreover, ultra-small superparamagnetic iron oxide NPs have been clinically validated to enhance MRI, allowing non-invasive monitoring of post-stroke inflammation by following macrophage recruitment into the ischemic brain [55]. CT imaging is also used for molecular imaging in stroke, even though it has less sensitivity than MRI [9]. In this context, gold nanoparticles conjugated with fibrin peptides have shown the ability to enhance image information on revealing brain vascular thrombus on CT imaging [56].

## 5. NPs for Stroke Treatment

Stroke lacks effective global therapy. This situation, nonetheless, is not due to a lack of potential drug candidates but rather to the inability of most of them to effectively cross the BBB. Several molecules have been reported to effectively relieve oxidative stress and inflammation-related stroke [57]. Nonetheless, their application is restricted by insolubility, short half-life, and low concentration in the brain [58], hampering their BBB permeation and their further accumulation on the ischemic tissue and thus preventing real translation to the clinic. NPs may address some of these challenges since they prolong the lifetime of drugs in living systems, they are appropriate vehicles for insoluble drugs, and some of the NP formulations are able to cross the BBB and induce neuroprotective effects by reducing the inflammatory response [59] or enhancing neurogenesis [60]. Thus, a great variety of NPs is currently under intense investigation (Table 1). NPs can reach the brain through different pathways, including paracellular and transcellular diffusion, efflux transport, or transcytosis methods such as carrier-mediated transport, receptor-mediated transcytosis, or adsorptive transcytosis [4,37,44].

The capacity of NPs to cross the BBB is dependent on the stroke phase. In the acute phase of stroke, the NPs can cross more easily the BBB associated with high permeability. In the late stages of stroke, the transport of the NPs through the BBB is impaired. In both cases, to improve brain targeting, the NPs are generally coated with biomolecules to target specific receptors in brain endothelial cells (see below). Several polymeric NPs formed by poly(butylcyanoacrylate) (PBCA), poly(lactic acid) (PLA), poly(lactic-co-glycolic acid) (PLGA), poly(amido amine) (PAMAM) dendrimers and chitosan have been used for BBB targeting [41,43,44]. Lipid-based NPs, such as liposomes, are also well studied to cross the BBB and have been proven to accumulate in the ischemic brain [61].

### 5.1. NPs in the Hyperacute Phase of Stroke

The hyperacute phase is key to patient management and final clinical outcome. Restoration of normal blood flow through recanalization is a critical step to achieve tissue survival and hugely depends on timing. The short IVT time window is a very limiting factor in stroke treatment and, although reperfusion is absolutely necessary for tissue survival, it may occasionally contribute to additional tissue damage being directly linked with HT [95]. The sudden and rapid reperfusion motivated by both tPA and EVT can cause the already wakened BBB to rupture. Moreover, plasminogen activator has effects on metalloproteinase activity, enhancing its activity and contributing to BBB disruption and hence its permeability increase [96] while mechanical clot removing through EVT implies, at least partially, direct endothelial trauma and potential BBB disruption [4]. Not surprisingly, NPs are emerging as a promising approach aiming to overcome the limitations of current treatments not only for improved and safer recanalization therapy but for rapid targeting of the hypoxia-related pathology of the disease.

Thrombolytic drug delivery requires NPs that are biocompatible, non-toxic, non-immunogenic, biodegradable, and prevent the rapid clearance of tPA by the immune system [97]. In this context, a variety of different NPs has been proven to efficiently deliver tPA to the thrombus in a safe and controlled way [9]. In fact, most of these strategies have shown the fibrinolytic effect of tPA to be potentiated while decreasing its associated risks. For example, t-PA loaded liposomes coated and targeted to the glycoprotein IIb/IIIa (GPIIb/IIIa) expressed in activated platelets have been proven to extend tPA half-life while increasing clot lysis and diminishing circulating fibrinogen, resulting in reduced HT risk when compared to conventional tPA treatment [65]. In the same line, polymeric NPs functionalized with fucoidan and directly targeting P-selectin of activated platelets also showed improved thrombolytic efficiency with no immunogenic risk [66]. Another strategy where tPA acts directly as a conjugated coating in polymeric NPs has been proved to efficiently target the fibrin in the thrombus producing clot lysis, even when using 10% of the typical dose, thus reducing tPA toxicity while extending its half-life and exerting neuroprotective effects [64]. Further strategies for tPA-targeted delivery, such as the use of an external stimulus to exert an effect on NPs, are being studied [9]. For example, the application of an external magnetic field can trigger liposomes to release tPA directly in the thrombus [98] or create local hyperthermia to accelerate thrombolysis through iron oxide cubes [99].

NPs loaded with neuroprotectants or antioxidants can be combined with tPA reperfusion therapy to reduce its negative effects [58,62,63]. For example, liposomes containing fausidil, a rho-kinase inhibitor able to prevent BBB disruption [100], showed neuroprotective effects ameliorating BBB disruption directly related to tPA when administered intravenously before conventional treatment [62]. Similarly, antioxidant catalase (CAT) and superoxide dismutase (SOD) loaded polymeric NPs (CAT/SOD-NP) have been shown to mitigate the inflammatory response and, importantly, inhibiting edema formation by protecting ROS-mediated BBB disruption when administered along with tPA [63]. NPs can also combine thrombolytic and neuroprotection drugs in the same formulation for maximum efficacy. For example, polymeric NPs loaded with ZL006 neuroprotectant coated with a platelet membrane and conjugated with thrombin-cleavable Tat-peptide-coupled rtPA were shown to selectively target the thrombus via the platelet membrane while the exposed Tat peptide allowed penetration across the BBB for ZL006e site-specific delivery. This approach showed not only efficient thrombolysis but also an evident decrease in ischemic area and reactive oxygen species level [67].

NPs can be used to regulate BBBP by the inhibition of metalloproteases (MMPs). It is known that MMP-9 is able to induce BBB leakage by interfering with brain endothelial cell tight junctions [101]. Thus, MMP-9 silencing and/or its direct inhibition could result in an interesting treatment for hyperacute stroke [69,70,71]. In this regard, NPs conjugated or loaded with an MMP-9 inhibiting peptide [69], tissue inhibitor of matrix metalloproteinases 1 (TIMP-1) [70], or CD147 antagonist peptide-9 [71] were able to cross the BBB and inhibit MMP-9 [69].

### 5.2. NPs and the Acute Phase of the Stroke: Tackling Neuroinflammation

In clinical practice, the vast majority of patients do not reach hospital care in the first 6 h, and thus therapeutic approaches for later phases tackling neuroinflammation and repair are essential in stroke care. This phase is mainly characterized by BBB disruption and inflammatory processes, which are motivated by the excitotoxic events generated at the beginning of the insult [4]. During this phase, the immune response has been described to be modulated by both innate and adaptive immune mechanisms. Initially, after sensing the tissue damage, the innate immunity acts fast, leading to inflammation. Subsequently, the already present inflammatory mediators lead to the activation and infiltration of inflammatory cells. The exact factors controlling the switch from innate to adaptive response are not completely identified [102]; nevertheless, it is known that the modulation of adaptive immune response plays an important role in the protection effect after stroke, offering therapeutic opportunities for the treatment of the disease [103].

Chemokines are described as participants on the initial immune response after pathologies such as stroke by promoting the initial migration of monocytes into the brain parenchyma, microglial response, and activation of defense genes response [104]. Microglia are responsible for the surveillance of the brain parenchyma, responding to the tissue damage and modulating the adaptive immune response. When microglia cells sense damage, mainly through Toll-like receptors (TLRs), they are able to switch their morphology from a ramified resting state, known as “M2” microglia and characterized by the anti-inflammatory modulation phenotype, to a more ameboid morphology known as “M1” microglia, characterized by producing cytokines and pro-inflammatory mediators for the induction of response to the detected damage. Within the other functions that microglia cells have, an important role has been reported in the maintenance of neuronal function, such as its involvement in the engulfment and remodeling of developing synapsis pruning during synaptic activity [105]. Microglia have been described to contact specialized areas of the neuron cell body and sense changes in neuron function and metabolic activity, as well as mitochondria functionality that protects neurons after acute brain injury obtained by an increase in the microglia processes that cover neurons and modulate them via the receptor P2Y12R [106]. An imbalance in microglia responses worsens injury and exacerbates pathological events during stroke [107,108,109], hence being a key target to avoid neuroinflammation after stroke.

After the initial activation of microglia, a key step for the modulation of the infarct, the regulation of the immune response becomes critical in the recovery of the ischemic tissue [103]. Whereas maintenance of hyperactivated microglia will induce an exacerbated inflammatory response, optimal microglia anti-inflammatory response will allow the tissue to respond to the injury, return to homeostasis and recover its functionality. Microglia cells can uptake higher proportions of NPs in their active state and thus are an interesting target for NP regulation [110,111]. Thus, nanoformulations aimed to target pathways associated with inflammation and modulation of microglia response can potentially induce a beneficial effect in responding to pathology such as stroke [59]. For example, EVs loaded with miRNA-126 were able to suppress microglial activation induced by an ischemic stroke in rats [72]. Brains treated with EVs showed significantly less TNF-α and IL-1b production than non-treated brains [72]. In a separate study, retinoic acid-containing NPs (more effectively than the free equivalent retinoic acid concentration) were able to suppress the inflammatory response of microglia cells (both in cell culture or in hippocampal slices) after exposure to an inflammatory agent such as lipopolysaccharide (LPS) [73].

### 5.3. NPs in the Subacute Phase: Targeting Angiogenesis

The subacute phase of ischemic stroke describes the regenerative events taking place around one week after stroke onset [4]. Among the restorative processes taking place during this phase, angiogenesis is key, contributing to the physiological, rather than pathological, BBB permeability increase and functional recovery after stroke. Mobilization of endothelial progenitor cells to the infarct site starts during the acute phase in response to the pro-inflammatory production of chemokines and growth factors, such as vascular endothelial growth factor (VEGF) and angiopoietin-2 (Ang-2) secreted by hypoxic cells [4]. Importantly, early induction of angiogenesis and upregulation of VEGF is correlated with an increased BBB permeability, which may increase the risk of HT. On the other hand, upregulation of VEGF and Ang/Tie-2 in later phases has been associated with a higher capillary density and enlarged vessel localization in the penumbra, with improving collateral circulation [17,112]. In the late subacute phase, tight junctions (TJs) are reorganized, with the help of sphingosine-1-phosphate and activated protein C [4], and consequently, the BBB permeability starts to decrease in the new vessels matured during the subacute phase and still under development. Nowadays, the treatment associated with the chronic phase is rehabilitation on a physical and cognitive level [113]. The positive outcome of this strategy is due to the fact that the neurovascular unit is restored, and neurogenesis is stimulated in the neurogenic niches in the SVZ of the lateral ventricles and in the subgranular zone (SGZ) of the dentate gyrus of the hippocampus. Many factors that contribute to the process are produced by astrocytes in the chronic phase, such as basic fibroblast growth factor (bFGF) and VEGF [114]. In particular, the bFGF is responsible for the increased expression of VEGF receptor Flk-1 in neural stem cells (NSC), and the neurotrophic factor VEGF induces the proliferation and migration of those cells [115].

As NPs offer an improved time window of drug activity, some interesting approaches are emerging on vascular protection and angiogenesis occurring in the timeframe of subacute stroke. Polymeric NPs are the most exploited for delivering angiogenic factors during the subacute phase of AIS [54,76,77]. Different polymers present specific physicochemical properties that are dependent on the properties of their building blocks. This allows versatile functionalization and drug conjugation of many polymer-based NPs. Some studies have shown the benefit of NPs to control angiogenesis during the subacute phase [75,76,77]. For example, the simultaneous delivery of stromal cell-derived factor (SDF)-1α and bFGF in the peri-infarct region by a hydrogel containing polymeric NPs promoted both neurogenesis and angiogenesis and significantly reduced the infarct volume [75]. In addition, the release of SDF-1α in the ischemic region by the dual-ionic pH-responsive copolymer, poly(urethane amino sulfamethazine) (PUASM), enhanced angiogenesis in the ischemic boundary zone after permanent middle cerebral artery occlusion (MCAO) [76]. Moreover, hypoxia-inducible factor (HIF)-1α loaded polymeric cationic NPs, surface coated with arginylglycylaspartic acid (RGD) peptide, exhibited an active role in the vascular regeneration as observed by visual analysis of the zebrafish, which allows easy experimental manipulation and an interesting model for studying cerebral ischemia and angiogenesis [77]. The therapeutic effect of this formulation was further examined on a rat model of ischemic stroke, showing significantly reduced infarct volume 3 days after treatment [77].

EVs have been used successfully to target the subacute phase of stroke. The therapeutic effect of EVs was first demonstrated after multipotent mesenchymal stromal cells (MSCs) systemically administered in a model of stroke were not able to reach the infarct area, but functional recovery was observed [90,91,116]. This has been associated with the paracrine effects of EVs by MSCs therapeutic factors, especially miRNAs, which play important roles in post-transcriptional gene regulation. MSC-EVs loaded with cholesterol-modified miR-210 showed promising data on improving angiogenesis for brain tissue repair after cerebral ischemia [78]. RGD-exo:miR-210 EVs administered once every other day for 14 days by intravenous administration in a transient MCAO mouse model reached the targeted lesion as given by significantly higher fluorescence intensity compared with the scrambled control [78]. Furthermore, the expressions of integrin β3, VEGF, and CD34 were shown to be significantly upregulated [78]. These results suggested a strategy for the targeted delivery of miR-210 to the ischemic brain and presented an angiogenic agent for the treatment of ischemic stroke. In a different study, rabies virus glycoprotein (RVG) was fused to the exosomal protein lysosome-associated membrane glycoprotein 2b (Lamp2b), which could efficiently deliver miR-124 to the infarct site [60]. Systemic administration of RVG-EVs loaded with miR-124 promoted cortical NSCs to obtain neuronal identity and protected against ischemic injury reporting robust cortical neurogenesis.

### 5.4. NPs in the Chronic Phase of Stroke: Promoting Neurorepair and Functional Recovery

After the first month, the chronic phase starts taking place. It is in this phase where true neurorepair mechanisms such as neurogenesis and vasculogenesis along with BBB repair take place; thus, therapeutic NPs administrated during the chronic phase of stroke should focus on delivering factors that can increase the NSCs proliferation, migration, and differentiation into neurons, in order to support and amplify the role of endogenously secreted factors [4], among which we can find growth factors such as bFGF, VEGF and brain-derived neurotrophic factor (BDNF), but also siRNAs and miRNAs. Therefore, the final outcome of the chronic phase, achievable also with the help of nanoformulations, is functional recovery.

NPs have been used successfully to promote neurogenesis both in vitro or in stroke animal models. For example, polyethylenimine (PEI) NPs loaded with retinoic acid, a neurogenesis and angiogenesis regulator, were able to enhance in vitro the proliferation program of human endothelial progenitor cells, which, in turn, secreted signals able to induce neuronal differentiation of NSCs [80]. PLGA NPs carrying miR-124 were able to induce neurogenesis in mouse NSCs cultured in vitro after oxygen and glucose deprivation but not in mice after photothrombotic stroke [86]. The dosage or other parameters of the treatment, such as timing and administration methodology, probably affected the in vivo neurogenic properties of the NPs. Yet, umbilical cord blood-derived endothelial progenitor cells transfected with PEI-encapsulated superparamagnetic iron oxide nanoparticles (SPION) loaded with HIF-prolyl hydroxylase 2 siRNA were able to reach the subventricular zone in photothrombotic focal ischemic stroke mice, proliferate and induce the neural differentiation of neural stem cells [79]. Finally, a hydrogel containing PLGA NPs loaded with cyclosporin A, a promising molecule for stroke therapy, induced the proliferation and survival of NSCs in stroke-injured rats [84].

Combinatorial release of biomolecules from NPs may regulate multiple regenerative pathways at the same time and thus improving the functional outcome. For example, two NP formulations loaded with epidermal growth factor or erythropoietin were entrapped in a hydrogel to induce the proliferation of NSCs and to limit cell apoptosis, respectively [82]. The NPs incorporated in the hydrogel released epidermal growth factor and erythropoietin (EPO) during 1 or 2 weeks, respectively. Moreover, PLGA NPs have been loaded with VEGF and Ang-1 to induce vascularization and axonal growth [85]. In this case, the NPs were introduced in a hydrogel functionalized with an antibody for Nogo receptor targeting to allow neuronal binding. Significant behavioral improvement was reported in MCAO mice 6 and 10 weeks after the stroke when the preferential use of the unaffected limb was reduced. Importantly, the release of multiple proteins from NPs can be regulated by the local expression of metalloproteases in the ischemic site [87]. Protein-based NPs that have been reacted with D- or L- type amino acids forming gels that responded to local levels of metalloproteases. The degradation kinetics of the gel was dependent on the composition of the peptides (i.e., formed by D- or L-type amino acids). The controlled release of VEGF in the mouse stroke cavity-enhanced vascularization and pericyte coverage in both the infarct and peri-infarct regions [87].

NPs modified with biomolecules to target receptors expressed in brain endothelial cells have enhanced accumulation in the brain after intravenous administration [43]. NPs containing neuroprotective agents (bFGF or bFGF plus small peptide inhibitor of caspase-3) and coated with an antibody targeting transferrin receptor-1 in brain endothelial cells showed the ability to cross the BBB and induce neuroprotection after intravenous administration [43]. Yet, neuroprotection was not observed when receptor-mediated transcytosis was inhibited with imatinib or when bFGF-loaded NPs were not conjugated with the targeting antibody. In a separate study, liposomes loaded with VEGF and modified with transferrin [88] were intravenously injected in rats and induced angiogenesis and improved neurological function compared to the controls 21 days after administration. Moreover, liposomes have also been modified to cross the BBB and target the infarcted area through the conjugation with T7 peptide and stroke homing peptide (SHp) [89]. Both these abilities were confirmed after the administration to MCAO rat models.

Biological NPs such as EVs are an alternative to synthetic NPs for the management of stroke at the chronic phase. One of the most explored sources for these EVs is the MSCs. EVs from this source were reported to induce neurogenesis in male rats 28 days after MCAO [90]. The treated animals showed increased axonal growth, doublecortin (DCX)-positive neuroblasts, and functional recovery. In a separate study, EVs from MSCs (both native or modified with miRNAs or small molecules such as enkephalin) administered in mice after cerebral ischemia improved neurogenesis and functional recovery relative to the control group [91,92,93]. In some of these studies, the EVs were surface-modified with biomolecules (e.g., transferrin) to target and cross the BBB [93]. EVs from other cell sources such as adipose-derived stem cells or urine have also been documented [72,94]. For example, urine-derived EVs were recently reported to induce NSCs differentiation in rats after systemic administration [94]. The positive effects in stroke models were suggested to be caused by the miR-26a that is responsible for the inhibition of the histone deacetylase 6.

## 6. Potential Harmful Effects of NPS and Nanotoxicity

So far in this review, we have discussed the advantages of using NPs, but while the nanomedicine field is very promising in stroke care and treatment, the specific interaction processes between biological systems and NPs are still uncertain [117], and thus, NPs could present toxicity risks when interacting with the human brain.

NPs toxicity or nanotoxicity refers to the ability of NPs to harmfully affect the normal physiology and structure of organs and tissues [117]. The final effect of nanotoxicity on the organism depends on the physiochemical properties of the NPs [117,118,119]. Regrettably, the unique characteristics that are responsible for the beneficial properties of NPs in medical applications, such as surface composition, particle size, and morphology, also lead to their toxicity [120].

First, the nanotoxicity of NPs may start as soon as they enter the circulation system due to a potential thrombotic response to contact with blood components. NPs could therefore induce the activation of platelets or any other factor influencing blood coagulation leading to thrombus formation [121]. This issue is of particular concern in stroke since this pathology is characterized by the presence of thrombus. Thus, the main aim in hyperacute care, which is thrombus dissolution, could be hampered by this nanotoxicity effect. Beyond this potential thrombotic response, NPs may become toxic when entering tissues [9]. In the particular context of stroke, we have mentioned a vast majority of NPs able to cross the BBB and reach the brain. This has great benefits for potential treatments, but the capacity to penetrate into the brain may subsequently influence BBB function and brain physiology and cause severe side effects due to nanotoxicity [122]. The nanotoxicity of NPs on the brain (neurotoxicity) seems to be related to an extensive production of ROS leading to oxidative stress and subsequently cytokine release, causing neuroinflammation [117,118,119,123]. Moreover, NPs may also have an immune system effect since the secretion of antibodies can act against NPs, limiting their use [124]. These are especially relevant points to have into account when developing NPs for the pathophysiological response in the acute phase of the stroke where the neuroimmune and neuroinflammatory responses are greater, and thus, this nanotoxicity effect could worsen the intrinsic neuroinflammatory acute response to stroke.

Another major problem of nanotoxicity in the brain is that some NPs may exacerbate BBB leakage. BBBP is one of the key processes in stroke development and, as such, is one of the main targets to account for in NPs development. In this context, it is therefore essential that NPs do not make blood vessels leakier than they already are, neither in the initial nor in the final phases of stroke. Moreover, even if NPs making vessels leakier could make them reach the brain more easily, this could motivate a later return to the circulation, causing hemolysis and the already mentioned platelet aggregation, among other adverse events [8]. Moreover, the nanotoxicity of NPs could affect neuron functioning, hampering neurorepair and neurogenesis in the subacute and chronic phases. The already mentioned oxidative stress due to free radicals’ production could not only lead to neuroinflammation but to subsequent apoptotic mechanisms, mitochondrial damage, and eventually neuronal death [122].

These harmful effects have been noted in different types of NPs [119]; nevertheless, those NPs that are metal-based appear to be the most cytotoxic [125]. Constrains with metal-based NPs are particularly important in the context of stroke since the ability of these NPs to cross the BBB reaching the brain tissue and their application in clinical imaging has gained interest in their use as drug delivery systems and diagnostic tools in this pathology. Due to their small size and specific physicochemical properties, metal NPs show a greater accumulation in the brain and induce higher toxicity than larger NPs [126]. Moreover, metal-based NPs are able to release metal ions due to their dissolution, exacerbating their toxicity [125]. For example, gold NPs are able to accumulate in the brain, inducing neurotoxic effects and increased seizure activity, cognition defects, and astrogliosis [119]. Furthermore, iron oxide NPs, very promising in stroke diagnosis through imaging, can interact with the brain cellular components, and depending on the presence, chemical composition, and charge of surface coating, they have the potential to alter synaptic activity leading to neuroinflammation, apoptosis, and immune cell infiltration [119,127].

All in all, it is evident that there is a lack of information on the potential neurotoxicity effects of NPs, which in turn makes it more difficult for their clinical translation.

## 7. Conclusions and Future Directions

Stroke is a complex, multifactorial and heterogeneous disease, and as such, it requires the search of different treatment approaches able to tackle the vast molecular processes occurring during its different hemodynamic phases. This review has discussed the most relevant NP approaches optimized for stroke management, from improved recanalization therapies and reduction in neuroinflammation in the hyperacute and acute phases to neurorestorative processes in the subacute and chronic phases.

New stroke treatments aiming to target ischemia are severely limited by the BBB. Therefore, specific drug delivery to the ischemic brain seems crucial in stroke treatment. In this regard, NPs acting as delivery systems have been gaining attention as the most promising approach for the potential delivery of therapeutic compounds. NPs can be synthesized from different materials, and their surface can be modified and functionalized through a vast variety of molecules to enhance their properties. This wide variability in design provides the NPs with specific properties such as selective targeting, high stability, and lack of immunogenicity [53], thus providing solutions to the limitations of conventional drugs such as sort-half-life in blood and low solubility enabling their BBB crossing [58].

In the context of stroke, understanding the biological and molecular process underlying its pathophysiology is key to develop new targeted drugs. For example, the BBB permeability increases in the first stroke phases could enable the facilitated delivery of compounds, while an intact BBB will need other transport mechanisms. Although promising, to date, none of these strategies have been applied to stroke clinical practice. While the nanomedicine field is very promising, NPs have several constraints that limit their clinical application. The risks associated with nanotoxicity leading to neuroinflammation and cell death or disruption of the BBB underline the need for the development of improved strategies for determining the neurotoxicity before NPs can reach human use. Moreover, the need for large clinical trials for the safety assessment of NP in patients limits their short-term translation to the clinic. With more and more NP platforms being explored, their approval could facilitate clinical translation as a novel implementation for already approve therapeutics [35], such as targeted recanalization through tPA loaded in nanocarriers.

As noted throughout this review, targeted approaches are mandatory to achieve therapeutic success; however, the mechanistic goals and delivery methods will have to vary over the time course of a stroke. Moreover, it is likely that a single intervention in a unique timepoint will not solve the complex pathology of stroke; nevertheless, the plasticity of NP formulation will likely be key in optimizing therapeutic approaches to the specific biological needs of each patient at each timepoint.

As a final remark, this work has aimed to report the fact that the design of NP as targeted drug delivery platforms could drastically change the landscape for stroke management from the improvement of current therapies to new approaches aiming to prevent and even restore the ischemic brain, all in all, resulting in better care and clinical development of the patients.

## Figures and Tables

**Table 1:** Nanoparticles for stroke treatment depending on the hemodynamic stroke phase.

Phase	Target	NPs	Payload	Outcome	Model	Ref.
**HYPERACUTE**	Rho-kinase	Liposomes	Fausidil	Protection against tPA harmful effects	SD MCAO rat	[62]
	ROS	Polymeric	Resveratrol	Protection against EVT harmful effects	SD tMCAO rat	[58]
		Biodegradable PLGA	CAT and SOD	Protection against tPA harmful effects	SD thrombo rat	[63]
	Fibrin	PEG-PCL	rtPA	Improved and no harmful reperfusion	SD MCAO rat	[64]
	GPIIb/IIIa of platelets	Liposomes with FGG C-terminal peptide	tPA	Improved reperfusion with no harmful effect	SD IVC trhombosis rat	[65]
	P-selectin of platelets	Polysaccharide-poly-IBCA + Fucoidan	rtPA	Improved reperfusion without harmful effect	Rat venous thrombosis	[66]
	TfR/GLUT receptor	Liposome dual-target nanocarrier	ZL006	Efficient trhombolysis and reduced cell apoptosis and ischemia	SD MCAO rat/ ICR mice	[67]
	MMP-9	Quantum dot nanoplexes	MMP-9 siRNA	ECM proteins upregulation and BBBP decrease	Human BMVEC/NHAs	[68]
		Amphibilic peptide	MMP-9-inhibiting peptide	MMP-9 inhibition	BBB model: hCMEC/D3 cell line	[69]
		Ps80-coated PLGA	TIMP-1	Early inhibition of MMP-9	In vitro: RBE4 / RBCEC+ astrocytes; In vivo: mice	[70]
		Polymeric NPs	CD147-antagonist peptide-9	Reduced brain infarct size and HT appearance	C57BL/6 tMCAO mice	[71]
**ACUTE**	Microglia activation	Adipose-derived stem cells exosomes	miR-126	Inhibition of microglial activation and inflammatory factors expression	MCAO rats	[72]
		Retinoic acid NPs	Retinoic acid	Reduction in microglia activation	N9 microglia cells; Organotypic hippocampal slices culture	[73]
	Transferrin receptor	PEGylated Selenium NPs	siRNA STAT3	Suppression of excessive inflammation and oxidative metabolism	MCAO rats	[59]
**SUBACUTE**	Stroke cavity	RGD-HA hydrogel	VEGF	Better angioenesis/establish axonal nets	Mouse MCAO	[74]
		PCN-NPs	SDF-1a, bFGF	Enhanced neurogenesis and angiogenesis	PTI	[75]
	Ischemic area	SDF-1-loaded micelles	SDF-1α	Enhanced neurogenesis and angiogenesis	Rat MCAO	[76]
	Integrin receptor	cRGD-dendrimer	N/A	Improved angiogenesis	PTI	[54]
		DMAPA-NPs	HIF-1α-AA plasmid	Enhanced angiogenesis, reduced infarct volume, and improved neurological function	Zebrafish AIS/Rat MCAO	[77]
		RGD-EVs	miR-210	Improved angiogenesis	MCAO mouse	[78]
	Neurons	RVG-EVs	miR-124	Enhanced cortical neurogenesis	PTI	[60]
**CHRONIC**	siRNA delivery/EPCs	Alkyl-PEI/SPIO	PHD2 siRNA	MRI/BLI tracking, Increased functional recovery, vascularization, neurogenesis, and Cxcr4 expression inducing cell mobilization and migration. Decreased infarct volume	In vitro: umbellical cord UCB EPCs In vivo: BALB/c nude mice	[79]
	Angio/neurogenesis	PEI	retinoic acid	NSC proliferation and differentiation, protection of ECs ischemic death	hEPC from stroke patients	[80]
	Neurovascular protection	PEI	miR-195	Improved neurogenesis, neuroprotection EC function/ less inflammation	In vitro: SH-sy5 In vivo: *tMCAO/MCAO rat*	[81]
	Sequential growth factor release	PLGA and PLGA/ poly(sebacic acid) NPs on HAMC hydrogel	EGF-PEG and erythropoietin	Controlled release of growth factor to the brain circumvents the BBB, neurogenesis	C57BL/6 murine stroke	[82]
	EPO dose reduction	PLGA	Erythropoietin	Effects of the EPO-NPs equivalent to 10 times the amount of free EPO	Unilateral AIS neonatal rat	[83]
	Increase efficiency of drug delivery	PLGA NPs in HAMC hydrogel	Cyclosporin A	Higher levels of CsA delivered with local injection, NSC survival, proliferation, and migration	Long-Evans endothelin-1 stroke rats	[84]
	Neural restoration via angiogenesis	PLGA NPs in a HA scaffold + anti-NOGO receptor antibody	VEGF and Ang-1	Behavioral improvement, vascularization, axonal growth	In vitro: HUAECs/ primary NSCs; in vivo: C57BL/6J MCAO rats	[85]
	Identification of new stroke therapeutics	PLGA	miR-124	SVZ neurogenesis, increased survival and neuronal differentiation of NSCs in vitro but no effects in vivo	In vitro: primary NSCs/ In vivo: C57BL/6 J PTI mice	[86]
	BBB crossing	Chitosan NPs + anti-tfR antibody	bFGF	Accumulation of NPs in brain parenchyma, neuroprotection	MCAO swiss albino mice	[43]
	Biomolecules delivery	Enantiomeric protein nanocapsules in HA hydrogel + RGD motif	VEGF and PDGF	Controlled release thanks to MMP-sensitive crosslinker, improved vascularization	C57BL/6 MCAO mice	[87]
	Increased brain delivery of VEGF	Liposomes functionalized with transferrin	VEGF	Neurogenesis, increased mRNA and protein VEGF, decreased infarct volume, functional recovery	SD MCAO rats	[88]
	Design of stroke dual-targeted lipososmes	liposomes conjugated with T7 peptide and stroke homing peptide (SHp)	neuroprotectant ZL006	BBB crossing, targeting of the ischemic area, improved neurological deficit, protection against apoptosis	In vitro: BCEC cells and PC-12 cells In vivo: SD MCAO rat and mice	[89]
	Stroke therapy with EVs	EVs from MSCs	N/A	Increased axonal density, functional recovery, neurogenesis, angiogenesis	MCAO Wistar rats	[90]
	MSC and MSC-EVs comparison	EVs from BMSCs	N/A	Improved motor coordination, neurogenesis, neuroprotection, angiogenesis	MCAO Mice C57BL6	[91]
	EVs’ study as therapeutics	Evs from MSCs	miR-133b	Motor recovery, neurite remodeling	In vitro: Primary neurons In vivo: MCAO rats	[92]
	Neurogenesis	EVs from BMSCs modified with transferrin	Enkephalin	Increased neuronal density, decreased p53 and caspase-3 levels	In vitro: primary neurons In vivo: MCAO rats	[93]
	Therapeutic effect of EVs from ADSC	EVs from adipose-derived stem cells (ADSC)	miR-126	Neurogenesis, angiogenesis, functional recovery	MCAO rats	[72]
	Effect of urine EVs on neurogenesis	Evs from urine	miR-26a	Proliferation and differentiation of NSC	MCAO rats	[94]

N/A = not available; SD = Sprague Dawley; MCAO = middle cerebral artery occlusion; tMCAO = transitient middle cerebral artery occlusion; ROS = reactive oxygen species; BMVE = microvascular endothelial cells; SDF-1α = stroma cell-derived factor 1; NHA = normal human astrocytes: FGG = fibrinogen gamma chain; IBCA = isobutylcyanoacrylate; EVT = endovascular thrombectomy; PLGA = poly(lactic-co-glycolic acid); rtPA = recombinant tissue plasminogen activator; IVC = inferior vena-cava; MMP = matrix metalloproteinase; ECM = extracellular matrix; BBB = blood-brain barrier; NPs = nanoparticles; HT = hemorrhagic transformation; PAA = polyacrylic acid; EV = extracellular veshicles.

## Data Availability

Data sharing not applicable.
